# The Mediterranean diet and cardiovascular disease

**DOI:** 10.1093/cvr/cvaf218

**Published:** 2025-11-10

**Authors:** María Barbería-Latasa, Miguel A Martínez-González

**Affiliations:** Instituto de Investigación Sanitaria de Navarra (IdiSNA), Navarra Institute for Health Research, 31008 Pamplona, Spain; Department of Preventive Medicine and Public Health, University of Navarra, Irunlarrea 1, Pamplona 31008, Spain; Instituto de Investigación Sanitaria de Navarra (IdiSNA), Navarra Institute for Health Research, 31008 Pamplona, Spain; Department of Preventive Medicine and Public Health, University of Navarra, Irunlarrea 1, Pamplona 31008, Spain; Biomedical Research Network Center for Pathophysiology of Obesity and Nutrition (CIBEROBN), Carlos III Health Institute, 28029 Madrid, Spain; Department of Nutrition, Harvard TH Chan School of Public Health, Boston

**Keywords:** Mediterranean diet, Cardiovascular disease, Review, UNATI, Alcohol and diet

## Abstract

Since the 1950s, when Ancel Keys popularized the traditional Mediterranean diet, an immense accrual of large and valid studies, including prospective cohort studies, randomized controlled trials (RCT), and systematic reviews, have been conducted to investigate the potential health benefits related to this dietary pattern. The available evidence shows that the traditional Mediterranean diet represents a sustainable, healthy, and highly palatable pattern with a wide array of associated health benefits. In particular, its protective effects on cardiovascular disease have been supported by several consistent large RCTs, namely, the Lyon Diet-Heart Study, PREDIMED, PREDIMED-Plus and CORDIOPREV. The traditional Mediterranean diet is not a vegetarian diet, but it emphasizes the preferential consumption of minimally processed plant-based foods: fruits, vegetables, whole-grain cereals, legumes, and tree nuts. Its unique components—different from other healthy, plant-based, food patterns—are extra-virgin olive oil as the main source of fat (keeping a high monounsaturated-to-saturated fat ratio), a high consumption of tree nuts, and low-to-moderate consumption of red wine with meals. However, the inclusion of an alcoholic beverage in a healthy diet is becoming increasingly controversial. An ongoing RCT, the University of Navarra Alumni Trialist Initiative (UNATI), aims to answer this question in 10 000 drinkers. In addition to conventional epidemiologic studies, metabolomics techniques have been applied to assess the role of adherence to the Mediterranean diet and to better understand the mechanisms involved in the protection afforded by this interesting dietary pattern. In this review, we update the available scientific evidence.

## 1. Introduction

It is well known that cardiovascular disease (CVD) is the leading cause of death worldwide.^1^ The World Health Organization (WHO) estimates that CVD claims 17.9 million lives each year. Not only does it contribute to increased mortality, but it is also a major factor in reduced quality of life.^1,2^ The prevalence of CVD has almost doubled since 1990. This increase also represents a large burden in terms of disability-adjusted life years (DALYs) and deaths, with a particularly severe impact on premature mortality. On the other hand, many risk factors are associated with CVD.^3^ These factors include lifestyle aspects, such as smoking, low-quality dietary patterns, low physical activity, and insufficient sleep quality, as well as various health metrics, mainly, plasma glucose levels, low-density lipoprotein cholesterol (LDL), blood pressure, and body mass index (BMI). In this context, the American Heart Association (AHA) proposed its ‘Life essential 8’ strategy of measures to reduce cardiovascular risk. Lifestyle changes, including smoking cessation, increased physical activity, 7–9 h of sleep per night, and adopting a healthy dietary pattern, are high priorities for preventive cardiology and cardiovascular health promotion.

## 2. Dietary patterns for the prevention of cardiovascular disease

Dietary patterns represent the state of the art in nutrition research. Rather than studying nutrients or foods in isolation, nutritional exposures should be analysed as a whole, by capturing the overall dietary pattern. Dietary patterns capture the quantities, proportions, variety, or combination of different foods, beverages, and nutrients in the diet, and the frequency with which they are habitually consumed. The concept of the dietary pattern provides a more comprehensive view of food and beverage intake. It is also more realistic than studying nutrients alone, because nutrients are not consumed in isolation but in combination. The study of dietary patterns has gained popularity in recent years. Food patterns represent the exposure of utmost interest for cardiovascular health because they are likely to be most strongly related to cardiovascular health outcomes, given the additive or synergistic effects of their components.

Low-quality dietary patterns are a priority for preventive cardiology not only because they are highly prevalent worldwide,^3^ but also because they are strong determinants of other cardiovascular-related health metrics, particularly body weight, blood pressure, and blood lipid and glucose levels. With respect to dietary patterns and cardiovascular health, the available evidence is solid and supports the adoption of overall healthy dietary patterns, such as the Dietary Approaches to Stop Hypertension (DASH), the Alternative Healthy Eating Index (AHEI), or the traditional Mediterranean diet (MedDiet) for the primary and secondary prevention of CVD. The main characteristics of these dietary patterns are summarized in *Table 1*. Of all the existing dietary patterns, the MedDiet, a tasty and highly palatable option, is the pattern most intensely assessed and the one with the strongest and most comprehensive evidence of its beneficial effects on cardiovascular health, including large and long-term randomized controlled trials (RCT) with hard clinical events as endpoints. These RCTs are available for both primary^4^ and secondary^5^ prevention.

**Table 1:** Main characteristics of beneficial dietary patterns

	AHEI^6^	DASH^7,8^	HPDI^9,10^	MedDiet^11,12^	MIND^13,14,15^
Approach	Dietary quality score	Reduced hypertension	Plant-based diet with avoidance of plant-based unhealthy foods	Traditional geographical dietary pattern	Brain Health Combines MedDiet and DASH
Main characteristics	High intake of fruit, vegetables, whole grains, fish, legumes and healthy fats. Limits intake of red and processed meats	Low sodium intake. High in fruit, vegetables, low-fat dairy products, whole grains, and lean protein. Limit sugar and saturated fats	Healthy plant foods (fruits, vegetables, whole grains, nuts, and legumes) Reduction in ultra-processed foods	High in olive oil, fruit, vegetables, fish, nuts, pulses, and whole grains diary. High monounsaturated:saturated ratio. Moderate intake of wine. Reduced in red and processed meat and sweets	Focus on green leafy vegetables and berries High in whole grains, nuts, fish, and olive oil Limit saturated fats and ultra-processed foods
Advantages	Improvement of cardiometabolic health	Reduction of blood pressure and lower cardiovascular risk	Reduced risk of chronic diseases and environmentally beneficial	Strong evidence of beneficial effects on cardiovascular, metabolic and neurological health	Prevention of cognitive decline and reduction of dementia risk
Disadvantages	Difficult to follow without dietary education as it does not specify quantities	Compliance with sodium restriction can be difficult for some individuals	Unplanned vegan diets can lead to deficiencies in certain micronutrients (B12, iron)	Some ingredients, such as olive oil, can be expensive particularly outside the Mediterranean region	Less scientific evidence than the Mediterranean diet

AHEI, Alternative Healthy Eating Index; DASH, Dietary Approaches to Stop Hypertension; HPDI, Healthy Plant-Based Diet Index; MedDiet, Mediterranean Diet; MIND, Mediterranean-DASH Diet Intervention for Neurodegenerative Delay.

In summary, the available evidence highlights the role of the MedDiet as an effective tool for the prevention and management of cardiovascular disease, making it a central strategy in preventive cardiology and precision nutrition.

## 3. The traditional Mediterranean Diet (MedDiet)

The traditional MedDiet is a dietary pattern that has been maintained for thousands of years and reflects the typical eating habits of the civilizations that inhabited the shores of the Mediterranean Sea, such as Greece (particularly Crete), Italy, Southern France, and Spain. Dwellers of these countries customarily consumed fresh, seasonal foods, such as olive oil, fruits, vegetables, nuts, legumes, breads and cereals (mostly unrefined), fish, and wine. In other regions of Lebanon, Morocco, Portugal, Tunisia, Turkey, and elsewhere in the Mediterranean basin, this food pattern was also followed with small variations.

Moderate alcohol consumption was common in non-Muslim Mediterranean countries in the context of the traditional MedDiet, but alcohol was not perceived as a psychoactive drug, but as part of the food pattern. It was mainly consumed in small amounts, adopting a characteristic drinking pattern, spread all over the week, with avoidance of any binging and in the form of wine consumed during meals. In fact, the ancient Greek word ‘symposium’ means drinking together with a group of friends.

Total fat intake in the traditional MedDiet was relatively high, even above 40% of total energy intake. In all instances, the ratio of the monounsaturated to the non-beneficial saturated fat was high, because of the high monounsaturated content of the liberally used olive oil.^16,17^

However, it was not until the 1950s and 1960s that an American researcher, Ancel Keys, popularized the term ‘Mediterranean diet’ with the Seven Countries ecological study.^16,18^ Keys and his team found that Mediterranean populations exhibited the lowest rates of CVD, despite their high fat intake. The main source of fat in the traditional MedDiet was olive oil, and its lipid profile of monounsaturated fatty acids is known for its beneficial bioactive properties; this is particularly true for extra-virgin olive oil (EVOO), which can account for more than 15% of total calories in a typical MedDiet. Another fluid aspect of the MedDiet is moderate wine consumption, particularly red wine during meals, which differentiates the MedDiet from other healthy food patterns.^19–21^

Since the MedDiet became popular, numerous epidemiological studies and some RCTs have been conducted to assess its potential beneficial health effects. In fact, it has become the most intensively studied dietary pattern worldwide.^16,17,21,22^ Particularly, over the last three decades, a large body of evidence has accumulated on the protective impact of the MedDiet on health, increased longevity, prevention of chronic diseases, and improved quality of life.^16,17,21–24^ The available evidence ranges from observational studies with long follow-up to clinical trials and numerous meta-analyses.

**Figure 1:**
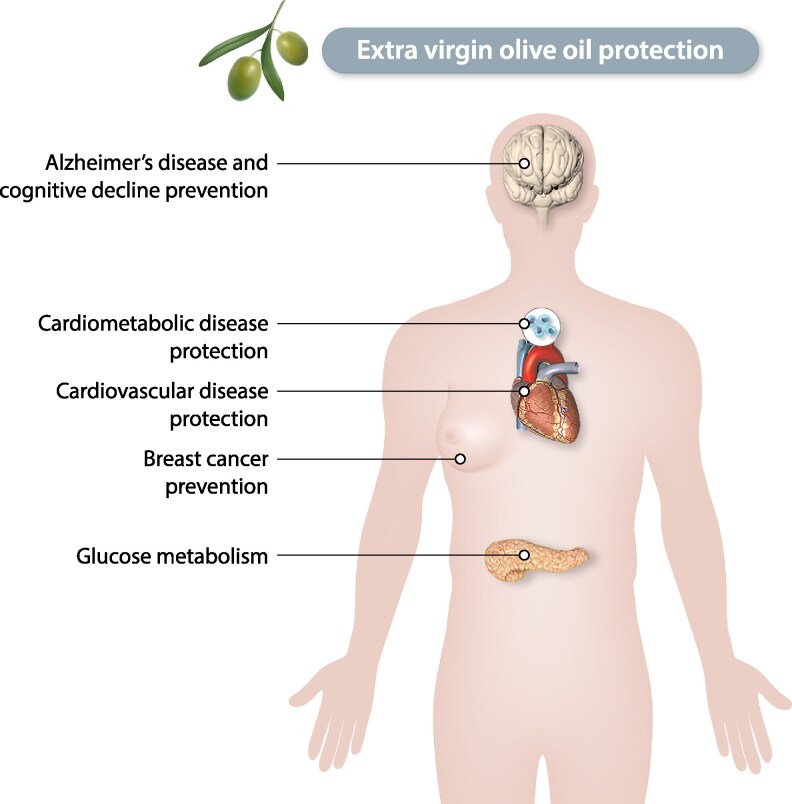
Extra-virgin olive oil principal benefits.

A high biological plausibility supports the protection afforded by the MedDiet throughout many avenues of previous research. For example, phenolic compounds and other antioxidants, which are abundant in Mediterranean foods (fruits and vegetables, nuts, legumes, olive oil, and red wine), exert anti-inflammatory effects with available evidence to strongly support their beneficial role against CVD and cancer (*Figure 1*). Also, monounsaturated fatty acid intake from olive oil has been consistently associated with a lower prevalence of risk factors. Furthermore, a higher dietary fibre intake is well-known to be associated with lower blood pressure and reductions in CVD risk. In addition, the frequent replacement of red and processed meats by fish and legumes, or the replacement of creamy and sugary desserts by fresh fruit (which is the usual dessert in the MedDiet), would be expected to bring about substantial reductions in risk factor burden.^25^

The traditional MedDiet, with its high consumption of vegetables and olive oil, is characterized by its high polyphenolic content. These compounds provide antioxidant and anti-inflammatory properties and several studies assessing the role of the MedDiet in cognitive function have been carried out in recent years. Both prospective observational studies^26–28^ and randomized controlled trials^29,30^ have found compelling beneficial associations of the traditional MedDiet with better cognitive function and a delay in cognitive decline. These effects seem to be explained by the antioxidant, anti-inflammatory, and cardioprotective properties of the MedDiet. The high presence of phenolic compounds helps to reduce oxidative stress in the brain, which delays the neurodegenerative process. Furthermore, improved cerebrovascular flow has been associated with an improvement in white matter hyperintensities^31^ and a reduction in subclinical cerebral infarcts.^32^

Therefore, antioxidant and endothelial-protective effects, together with stimulation of neurogenesis, synthesis of neurotrophic factors or modulation of neuronal signalling,^33^ may contribute to improved global cognition and reduced memory decline.^29^

The MedDiet has been shown to exert many health benefits in the adult population. However, there are fewer randomized trials in the paediatric population, and the evidence is more limited.^34^ This scarcity of scientific evidence is not limited to the MedDiet but also extends to other dietary models. But for the MedDiet, some trials reported benefits in children. Those studies found significant reductions in body mass index (BMI) and adiposity in children and adolescents who presented a higher conformity with the MedDiet.^35^ Similarly, a recent systematic review reported statistically significant beneficial changes in cardiometabolic biomarkers with the MedDiet intervention in children and adolescents.^36^ That recent systematic review found significant reductions in systolic blood pressure, triglycerides, total cholesterol, and low-density lipoprotein cholesterol and a significant increase in high-density lipoprotein cholesterol in children with a MedDiet intervention as compared to a control diet. Therefore, the MedDiet not only appears to be safe in children and adolescents, but it can also be recommended for its benefits, given that preventive cardiology interventions should be implemented early in life.^37^

A further place of honour for the MedDiet was its recognition by the UNESCO in 2010 as an intangible cultural heritage for the benefit of human health.^17,38^

However, a pending question is the transferability of the benefits of the traditional MedDiet pattern outside the Mediterranean area. Can they be transferred to other populations, where olive oil consumption has generally been low? The facts are that the largest available trials have specifically used olive oil,^4,5^ and olive oil consumption is growing in many other parts of the world. Beyond the boundaries of the Mediterranean basin, a wide and diverse range of culinary practices and cuisines from many cultures and other geographical regions of the world are compatible with healthiness and can be fostered with the aims of health promotion. The main building stones of the MedDiet can be incorporated into these diverse food cultures, notably, the Asian cuisines, for example, the traditional Okinawa diet, other Japanese food practices, and several ‘blue zones’,^39^ without compromising their unique identities. A cross-cultural approach (sometimes referred to as ‘fusion diet’) merging the healthy elements from different customs and traditions can be seen as an optimal strategy. There is a need to emphasize commonality over differences and to promote acceptance across populations with different food traditions and culinary backgrounds. This approach will enhance long-term adherence in many populations’. Therefore, the so-called ‘Mediterranean-type’ dietary patterns using other alternative unsaturated oils may become more similar to the real MedDiet by eventually adopting the use of olive oil.^40^

## 4. Limitations in the definitions of the MedDiet

The most commonly used indexes for defining the MedDiet (*Table 2*) are the Mediterranean Diet Score (MDS), the Mediterranean Diet Adherence Screener (MEDAS), and the Alternative Mediterranean Score (aMED).

**Table 2:** Mediterranean diet scores

	Mediterranean diet score (MDS) (Trichopoulou, 0–9 points)	Mediterranean diet adherence screener (MEDAS) (PREDIMED 0–14 points)	Alternate Mediterranean diet score (aMDS) (0–9 points)
Positively weighted components	Monounsaturated/saturated^a^ Vegetables^a^ Fruits and nuts^a^ Legumes^a^ Fish^a^ Cereals^a^	Olive oil as main culinary fat ≥ 4 tablespoonc/d olive oil ≥ 2 servings/wk olive oil sauce with tomato, garlic, onion, or leek (‘sofrito’) ≥ 2 servings/d vegetables ≥ 3 servings/d fruits ≥ 3 servings/wk nuts ≥ 3 servings/wk legumes ≥ 3 servings/wk fish Preference for poultry or rabbit > red or processed meats	Monounsaturated/saturated Vegetables (no potatoes) Fruits Nuts Legumes Fish Cereals (whole grain)
Negatively weighted components	Meat/meat products^b^ Dairy products^b^	<1/d red/processed meats<1/d butter/margarine/cream<1/d carbonated/sugar-sweetened beverages<2/wk commercial bakery, cakes, biscuits, or pastries	Meat/meat products
Alcohol	5–25 g/d (women) of wine 10–50 g/d (men)	≥ 7 glasses/wk of wine	Moderate

^a^One point if the consumption was at or above the sex-specific median.

^b^One point if the consumption was below the sex-specific median.

The MDS proposed by Trichopoulou^22^ is one of the most widely used scores in nutritional epidemiology to assess adherence to the MedDiet. With a maximum score of 9, it positively assesses (by splitting consumption by the median of the sample) frequent consumption of vegetables, fruits and nuts, legumes, whole grains, fish, and olive oil (as the ratio of monounsaturated to saturated fat). Moderate consumption of alcohol (as red wine with meals) is also considered beneficial. On the other hand, high intakes of meat (or meat products) and dairy products (also dichotomized by median consumption) have a negative impact on this score.

The aMED is a score designed for non-Mediterranean populations. It is somewhat more flexible in aspects such as olive oil and allows for other unsaturated fats to make it more applicable to non-Mediterranean countries, particularly the USA.

The MEDAS was developed specifically for the PREDIMED trial^4,22^ and scores adherence up to a maximum of 14 points. It is quick and easy to use for intervention studies. It scores positively on the use of extra-virgin olive oil as the main source of fat, the consumption of at least two daily portions of vegetables and three portions of fruit, frequent consumption of nuts, legumes, and fish, and moderate consumption of wine with meals. It also includes a low intake of meat, commercial bakery products, butter and cream, sodas, and processed foods. A score of 10 or higher indicates high adherence to the diet.

Although the MedDiet is the dietary pattern with the largest epidemiologic accrual of beneficial evidence, and even though several large RCTs have been successfully completed, some criticisms were raised on the results of these trials and also on well-conducted prospective cohort studies in relation to the different scoring systems.

One of the main issues is that there is no uniform consensus on the exact definition of the MedDiet. On the one hand, the available studies used different cut-off points in the scores to assess adherence, and on the other hand, these cut-off points very often depend on the particular characteristics of the assessed sample. It is true that the use of medians (or other quantiles) of consumption of each food group or nutrient in the sample population is a common practice in nutritional epidemiology when deriving dietary adherence scores. However, this procedure poses a certain problem because it depends on the sample characteristics, thus hindering between-study comparisons and the generalizability of results.^41^

## 5. Limitations in the available systematic reviews

Although there were differences between studies in the medians (or other quantiles) of consumption for each food group or nutrient used to derive the scores, the striking similarity in the protective results found for the MedDiet in different populations provides strong evidence for the consistency of the effects, regardless of the specific cut-off points, as shown by a large number of published systematic reviews and meta-analyses,^42–56^ also when assessing the effects on inflammation and endothelial function.^57,58^ Furthermore, most of the available dietary analyses have used the food frequency questionnaire, which is a better tool for ranking participants according to relative consumption levels within the studied sample, rather than for measuring absolute intakes.

Some systematic reviews and meta-analyses have been criticized for potential methodological limitations. For example, they included a fewer number of original studies than the number of already published reviews.^59,60^ Admittedly, some meta-analyses also used suboptimal methods to draw their conclusions, or they did not consider potential moderators, nor they did sufficiently deep analyses to identify and explain potential sources of heterogeneity. However, some of them were of good quality and their results were consistent with the other systematic reviews, with only rare and isolated exceptions.^61^

Many studies have been conducted by researchers born in the Mediterranean area with a possible nostalgic bias. Frequently, they studied a relatively homogeneous population by including only participants from Mediterranean countries. However, it should be noted that the first researcher to study the traditional MedDiet, who coined the term, was not Mediterranean, but American.^62^ Furthermore, in recent years, a plethora of studies have reported protection by the MedDiet or by its unique components in non-Mediterranean populations.^16,17,40,42–56,63^

## 6. Limitations in the available randomized trials

Finally, some authors have pointed out the limitations of some RCTs conducted to assess the effects on hard clinical endpoints caused by interventions with the MedDiet.^64^ But their criticisms have been refuted with new evidence^5,21,22,25^ reinforcing previous findings.

The Lyon trial was a secondary prevention trial in which patients with CVD were randomized to MedDiet intervention or control (a low-fat-like diet). This trial found a reduction in mortality and a lower recurrence of myocardial infarction as compared with the control group.^65^ However, the intervention was not exactly equivalent to the typical and traditional MedDiet. The percentage of monounsaturated fatty acid (MUFA) intake in the intervention group was lower (12.9%) than the usual 15–20% MUFA coming from olive oil in traditional MedDiets. In addition, there was no pre-specified protocol to determine under which scenarios the trial should be stopped, so stopping the trial earlier than planned could have led to an overestimation of the effect. Finally, actual dietary changes were not completely recorded.

Another RCT is the well-known ‘PREvención con DIeta MEDiterránea’ (PREDIMED) trial, which is the largest trial to date to assess the effects of the MedDiet on CVD. This was a Spanish primary prevention trial in which 7447 men and women at high cardiovascular risk (55–80 years, 57% women) were randomized into 3 groups. In the two active intervention groups, participants were allocated a MedDiet with free provision of EVOO (1 L/wk for the whole family, with the goal of consuming 45 g/d) or to a MedDiet and free provision of nuts (to consume 30 g/d). The control group received non-food gifts and advice to reduce all sources of fat. As in the Lyon study, the intervention had to be stopped prematurely by advice of the independent Data and Safety Monitoring Board, 4.8 years after the start of the trial, instead of the planned 6 years. This was due to early strong evidence of benefit, showing hazard ratios of 0.70 [95% confidence interval (CI), 0.54–0.92] and 0.72 (95% CI, 0.54–0.96) for the MedDiet with EVOO and MedDiet with nuts groups, respectively, compared with the low-fat control group.

In contrast to the Lyon trial, PREDIMED had a protocol that was drawn up before the start of the trial, detailing when and under what conditions the study should be stopped. However, in 2017, isolated voices criticized the randomization criteria and questioned the results obtained.^64^ The PREDIMED team re-analysed the results. The original paper was withdrawn by the investigators and replaced by another version, which took into account the potential departures of individual randomization in a small subset of participants and conducted a wide array of new sensitivity analyses. They showed that there were no changes to the previously published results with even better results after excluding members of the same household and other very small groups randomized as a cluster (rather than individually) at only one of the 11 centres. The analyses after these exclusions (*Table 3*, last row of the republished paper in 2018) showed a hazard ratio of 0.65 (95% CI, 0.50–0.85) for the two MedDiet groups combined as compared with the low-fat control group.^4^ Moreover, this republication included a supplement of almost 100 pages with many ancillary analyses that strengthened the robustness of these findings, supporting a substantial protection by the MedDiet against the primary CVD endpoint (a composite of nonfatal myocardial infarction, nonfatal stroke, or any cardiovascular death). In addition, the per-protocol analysis (considering actual adherence) showed a hazard ratio for the primary endpoint of 0.42 (95% CI, 0.24–0.63) for the MedDiet as compared to the control diet.

**Table 3:** Summary of the main results of the Mediterranean diet randomized trials

CLINICAL TRIAL	Lyon trial	PREDIMED trial	PREDIMED plus trial	CORDIOPREV trial
Recruitment (years) and intervention	1988–1997 4 years	2003–2011 4.8 years	2013-ongoing 6 years	2009–2021 7 years
Country	Lyon, France Single centre	Spain Multicentre	Spain Multicentre	Córdoba, Spain Single centre
Participants	605	7447	6874	1002
Type of trial	Secondary prevention	Primary prevention	Primary prevention	Secondary prevention
Intervention groups	Type of MD with special margarine Low-fat diet	MD + EVOO MD + mixed nuts Low-fat diet	Energy restricted MD + PA MD	MD Low-fat diet
Primary outcome	Miocardial infarction recurrence and CV mortality	CV events	CV events	Recurrence of CV events
Main result	70% Reduction of recurrence and CV mortality	30% Reduction of CV events	ongoing	26% Global reduction 33% only men

MD, Mediterranean diet; EVOO, extra-virgin olive oil; PA, physical activity; CV, cardiovascular.

Finally, the results of PREDIMED were basically replicated in a secondary prevention RCT also conducted in Spain with 1002 participants (20–75 years, 17.5% women). This important RCT is known as CORDIOPREV.^5^ In CORDIOPREV, patients with established coronary heart disease were randomized to a MedDiet supplemented with EVOO or to a low-fat diet (control group) and followed for 7 years. The protocol for the intervention was almost identical to the PREDIMED, except that there was no MedDiet + nuts arm. All participants were individually randomized, and the multivariable-adjusted hazard ratios of the different models ranged from 0.72 (95% CI: 0.54–0.95) to 0.75 (0·57–0·998) in favour of the MedDiet.

Overall, despite the possible (and remote) limitations discussed above, and the more realistic (and always challenging) issue of attaining perfect compliance with the intended dietary changes (particularly at advanced ages), the MedDiet showed a sufficiently strong protection in these RCTs. These findings are also surrounded by a wide range of prospective cohort studies with good control for confounding conducted in different parts of the world, which showed even greater protection. The Mediterranean food pattern has the added advantage of being highly tasty and it has also been used for millennia without causing any health damage. In fact, life expectancy in many Mediterranean countries has usually been among the highest in the world.

## 7. The Mediterranean lifestyle and the PREDIMED-plus trial

The MedDiet is not just about food; it is a cultural tradition that involves more than just food. It is about the way the food is cooked and consumed, and also includes other aspects of the lifestyle beyond food consumption.

**Figure 2:**
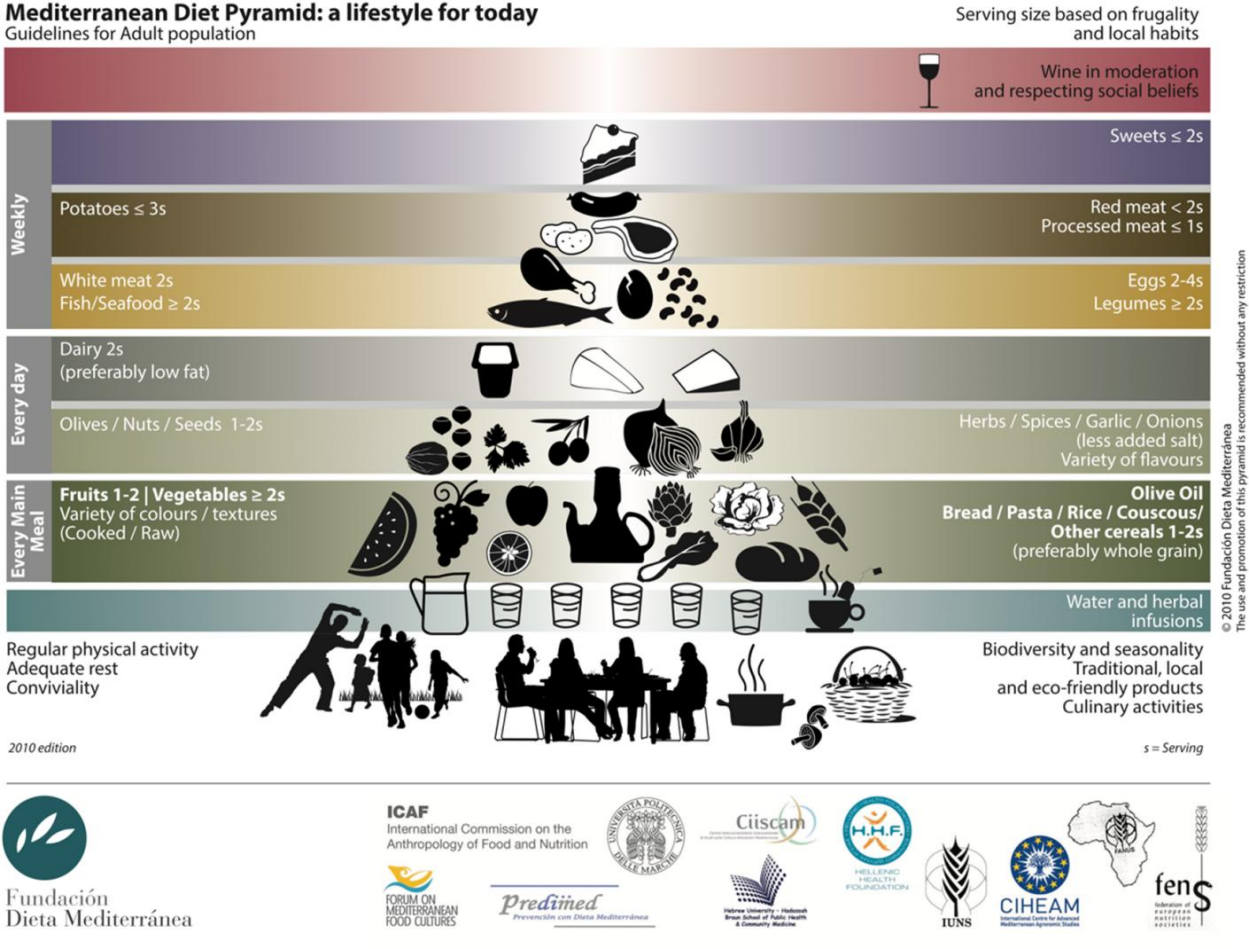
The Mediterranean diet pyramid.

As shown in *Figure 2*, the MedDiet Pyramid^26^ adds water consumption, physical activity, adequate rest, family meals, and conviviality. Priority should be given to seasonal and local foods, with respect for the environment and the preservation of traditional cooking techniques.

In this line of thought, PREDIMED-Plus is a multicentre, single-blind, randomized trial conducted in 23 Spanish centres with the aim of assessing other aspects, such as keeping a healthy weight, eating more frugally, and maintaining an active lifestyle. It was originally funded by an Advanced Research Grant of the European Research Council. Between September 2013 and December 2022, 6874 men aged 55–75 years and women aged 60–75 years without cardiovascular disease but with a BMI of 27–40 kg/m^2^ and ≥3 criteria for metabolic syndrome were recruited. They were randomized to an intervention group with a calorie-restricted MedDiet, physical activity, and behavioural support, or to a control group with a MedDiet without any calorie restriction or recommendations for weight loss or physical activity beyond usual care. Participants in the intervention group had monthly contacts for the first year and specific targets for weight loss through reductions in calorie intake and increased physical activity. This intervention meant a MedDiet with further restrictions on the consumption of red and processed meat, soda drinks, butter and margarine. They were also advised not to add sugar to drinks and to limit refined cereals especially, white bread, which is the most common staple food in Spain. The primary endpoint is a composite cardiovascular event identical to that in PREDIMED. The active intervention of the PREDIMED-plus trial has been ongoing until the end of 2016, and then, by recommendation of the Data and Safety Monitoring Board, two additional years of only observation were added.

Preliminary results of PREDIMED-Plus indicate that participants on the calorie-reduced MD achieved significantly better adherence to the intervention at 12 months and improvements in the main cardiovascular risk markers (BMI, weight, waist circumference, most lipid fractions, and blood pressure), which could lead to a positive impact on cardiovascular health.^66^ Furthermore, type 2 diabetes was substantially reduced by the intervention.^67^ Overall, it seems clear that dietary and lifestyle interventions in these high cardiovascular risk groups are feasible and result in clinically meaningful changes.

## 8. Criticisms on including wine or ethanol in the Mediterranean diet

Wine, together with olive oil, is a unique component of the MedDiet. It should be noted that the Mediterranean alcohol drinking pattern refers not only to the amount of ethanol consumed, but also to the consumption of alcoholic beverages according to a specific pattern.^68,69^ This pattern of moderate alcohol consumption can be traced back to the traditional habits of the populations of Greece, Italy, Southern France, and Spain. It consists of low-to-moderate consumption of red wine with meals, with a consumption spread over the week, always with meals, and avoidance of binge drinking. Also, as part of the drinking pattern, it included red wine as the preferred beverage and the avoidance of distilled beverages. These seven aspects defined the Mediterranean drinking pattern and have been associated with lower all-cause mortality in several studies, as well as lower incidence of CVD and hypertension.^68,69^

Red wine is the characteristic alcoholic beverage of the MedDiet and is characterized by the bioactive phenolic compounds present in the grape skins, which give it a high anti-inflammatory capacity. These properties add to the effects found in other alcoholic drinks, such as platelet anti-aggregating capacity, HDL increase, and improved insulin sensitivity.^70^

However, according to the International Agency for Research on Cancer, alcohol was classified as a type 1 carcinogen. It is estimated that 4.1% of cancer cases can be attributable to alcohol. Alcohol consumption has been causally associated with the development of seven types of cancer: esophageal, pharyngeal, laryngeal, oral cavity, liver, female breast, and colorectal. For breast cancer, the risk increases at low levels of consumption, so there appears to be no safe minimum level of alcohol. In fact, the World Health Organization currently promotes abstinence from alcohol, with no safe or recommended intake.

Regarding CVD or the total global burden of disease, the abstinence recommendation is supported by modelling global assessments and Mendelian Randomization (MR) studies. However, these studies also have some limitations and are based on several assumptions that are not always verifiable, and make them neither comparable nor a substitute for a properly conducted RCT.

Limitations of MR studies include the lag between the time of pseudo-randomization and the time of eligibility and outcome recording, pleiotropy, inconsistency between different outcomes, or the degree of heterogeneity in alcohol consumption between categories of the assumed scores based on genetic (or even geographical) information. Also very important is the inability of MR and global modelling studies to capture the type of beverage, and the actual drinking pattern (frequency, distribution over the week or weekend, consumption with or without meals, avoidance of binging and other potential effect modifiers of alcohol intake). All these aspects are very likely to act as important effect modifiers, beyond the total amount of ethanol ingested.

All these issues highlight to the need for conducting a randomized trial. The fact is that MR is unlikely to say the last word. In any case, it should not be forgotten that in 2009, a Greek study found that the removal of the moderate ethanol item from the MDS resulted in a loss of 23.5% of the protection provided by the MedDiet against all-cause mortality, making it the most relevant item in that study.^71^ Similar reductions have been found in other well-conducted cohort studies that applied appropriate methods for the control of confounding.

Ethanol is a psychoactive substance and its use in the young population tends to be associated with binge drinking, which involves the consumption of large amounts of alcohol, usually distilled, in a short period of time. In addition, the main causes of death in this age group are closely linked to alcohol: traffic accidents, injuries, suicides, and breast cancer in women. These harmful effects of alcohol are particularly relevant in the population aged 20–35 years where there is usually no relevant burden of coronary heart disease or stroke that can be theoretically prevented by low-to-moderate alcohol consumption. Therefore, in subjects younger than 35 years of age, there will be no basis for any protection from alcohol consumption, and in this group, the recommendation should undoubtedly be total abstinence.^69^

For the population older than 50 years, many voices also advocate abstinence and zero alcohol as the only safe alcohol intake. On the other hand, the recommendation for adult drinkers of this age range to drink in moderation (≤7 drinks per week for women and ≤14 drinks per week for men) can be seen as a harm reduction option, provided that it is also accompanied by the recommendation to avoid binge drinking and to drink only with meals. In this population, particularly if they are at high risk of CVD, it appears that following a Mediterranean drinking pattern may reduce the risk of CVD and premature death.^22,68,69^

However, recommendations supporting the benefits of low-to-moderate alcohol consumption are based only on observational studies, with limitations for causal inference and, especially, a threat to be affected by the so-called ‘sick quitter effect’ or by the ‘never starter sick’. These possible biases can compromise the validity of conventional observational studies if abstention is due to pre-existing disease.

Alternative views supporting blanket abstinence for all are based on these threats and on modelling and MR studies. But these last studies might also have serious limitations for a firm causal inference. As mentioned above, an RCT in drinkers older than 50 years should be conducted to solve this controversy. In any case, it should be stressed that, according to the precautionary principle, people in this age group who are abstainers should never be advised to start drinking alcohol as a supposed means for cardiovascular prevention.

## 9. A trial testing alcohol abstention vs. the Mediterranean alcohol pattern

**Figure 3:**
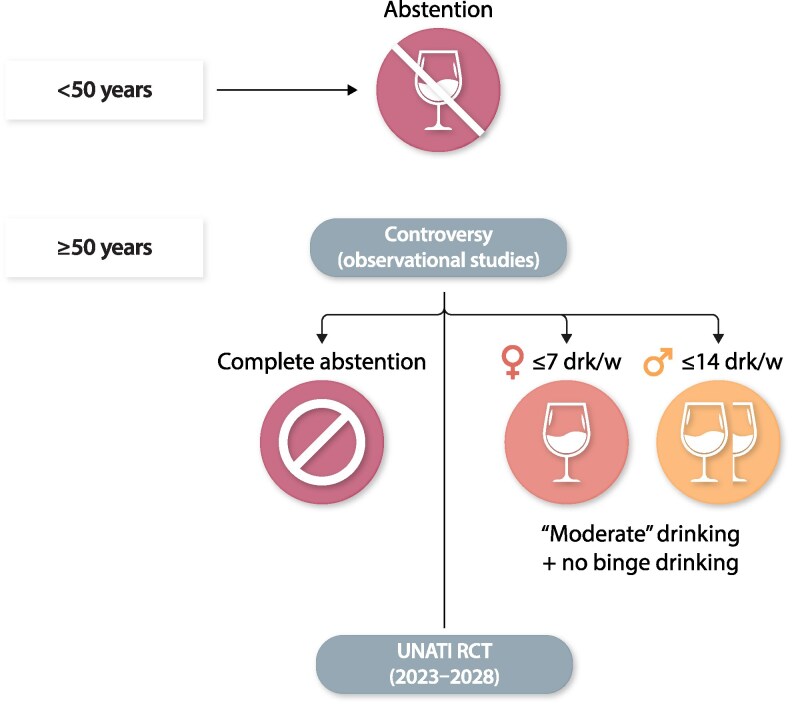
Controversy on alcohol recommendations.

**Figure 4:**
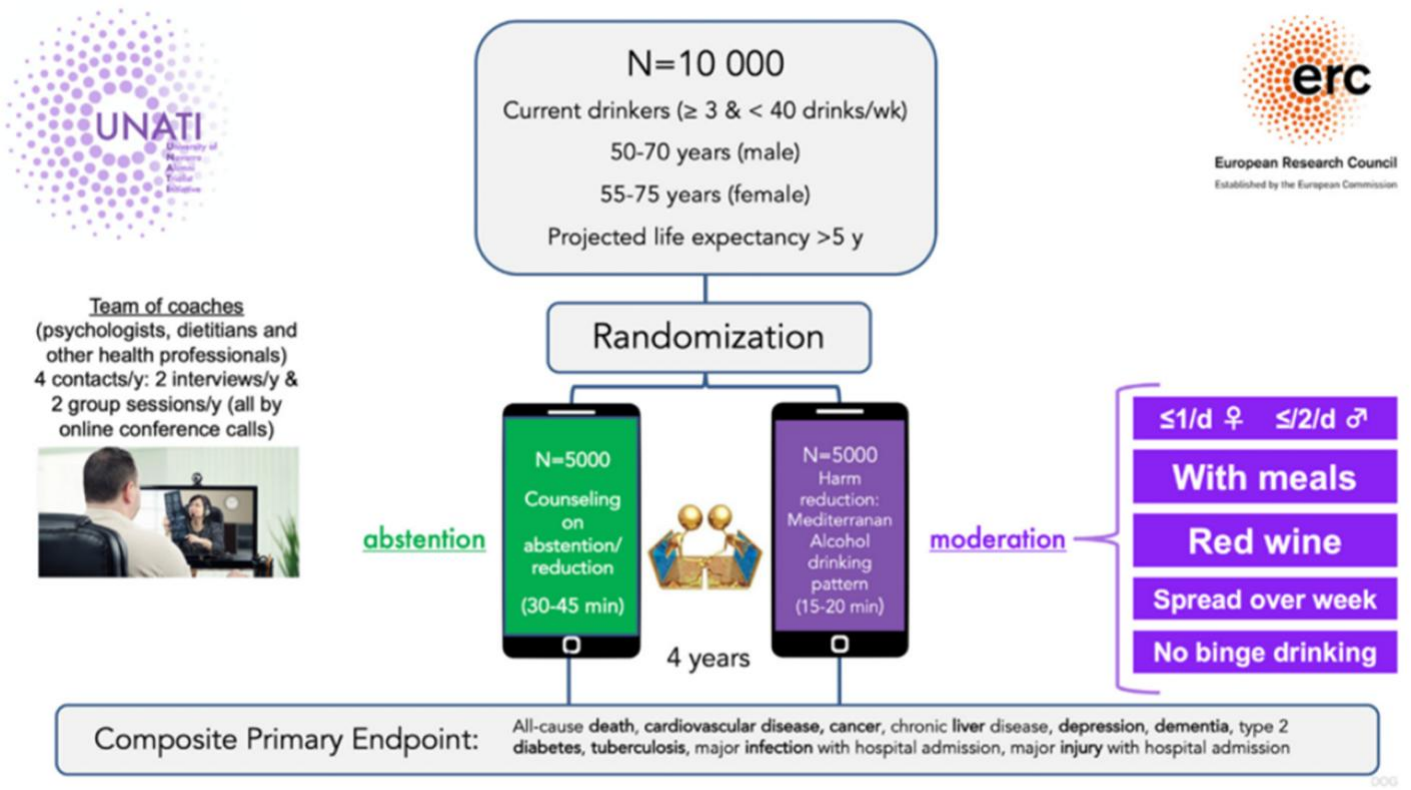
Randomization process for UNATI participants.

The European Research Council (ERC) has recently funded a large trial on the effects of alcohol cessation to be conducted at the University of Navarra (Spain). The name of this trial is UNATI (University of Navarra Alumni Trialist Initiative). It is a randomized non-inferiority trial, and it will be the largest randomized alcohol trial in the world to date, which aims to provide solid evidence on the question of alcohol cessation in drinkers over 50 years of age, where recommendations are controversial (*Figure 3*). The intervention will be delivered via conference calls with quarterly contacts over 4 years (*Table 4*). Recruitment started in 2024 (up to Novemberr 2025, there were >7500 recruited and randomized participants). The UNATI trial includes men (50–70 years) and women (55–75 years) who usually drink between 3 and 40 units of alcohol per week and are living in Spain (*Figure 4*). This non-inferiority trial aims to recruit more than 10 000 participants who will be randomized to receive 2 different types of advice: total abstinence or moderate alcohol consumption according to the Mediterranean drinking pattern.^12,46^ The primary outcome is a composite of total mortality, invasive cancer, major cardiovascular events (nonfatal myocardial infarction, nonfatal stroke, cardiovascular death, heart failure, or atrial fibrillation), liver cirrhosis, type 2 diabetes, depression, dementia, injury with hospitalization, or infection with hospitalization. Results are expected after 2028.

**Table 4:** Questionnaires during the follow-up period in the UNATI trial

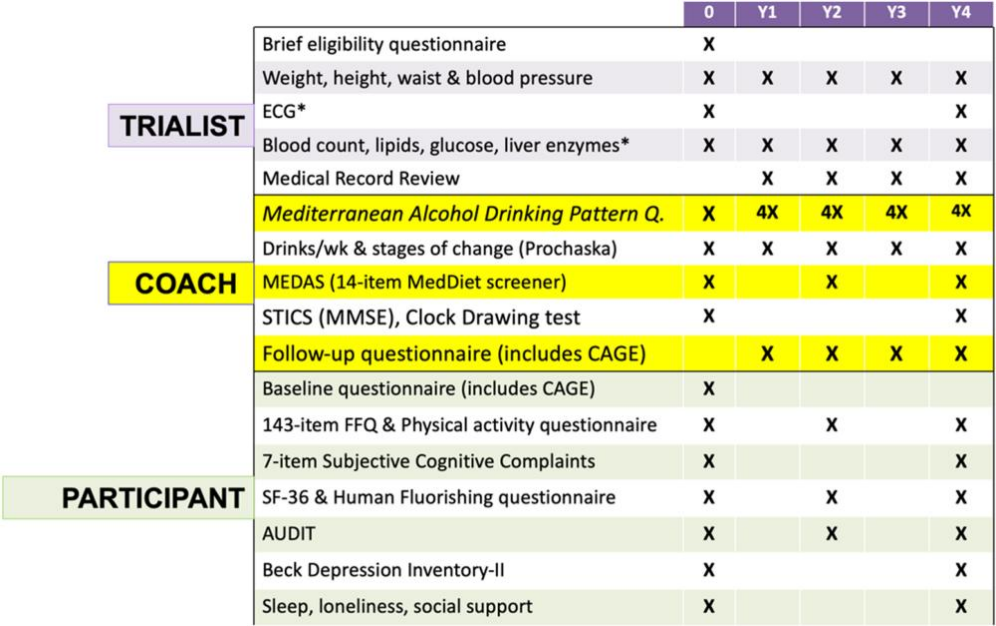

The Mediterranean Alcohol Drinking Pattern Questionnaire will be repeatedly assessed 4 times/y (4x), every 3 months, and it will provide an excellent opportunity for the coaches to conduct a negotiated agreement with the participants in the moderation in order to upgrade their adherence to the Mediterranean Alcohol Drinking Pattern and to agree goals for the next 3 months.

STICS (MMSE), Telephone adaptation of Mini-Mental Status Examination (MMSE); CAGE, alcohol misuse screening test; AUDIT, alcohol abuse screening test; SF-36, Questionnaire of quality of life Short-Form 36 (SF-36); FFQ, validated food frequency questionnaire (143 items); MEDAS, Mediterranean Diet Adherence Screener (14 items).

^a^ECG and analytical data are only collected by the trialist from the clinical records if they were done according to the clinical situation of the patient.

## 10. Metabolome-based studies on the MedDiet and CVD prevention

In recent years, computational advances in high-throughput chemical technologies, i.e. metabolomics and other -omics, have opened the door to precision medicine. One of the aims of -omics research is to identify significant associations between dietary metabolites and disease. Metabolomics is the study of the chemical transformations that take place in cells or organisms involving small-molecule metabolites.

**Figure 5:**
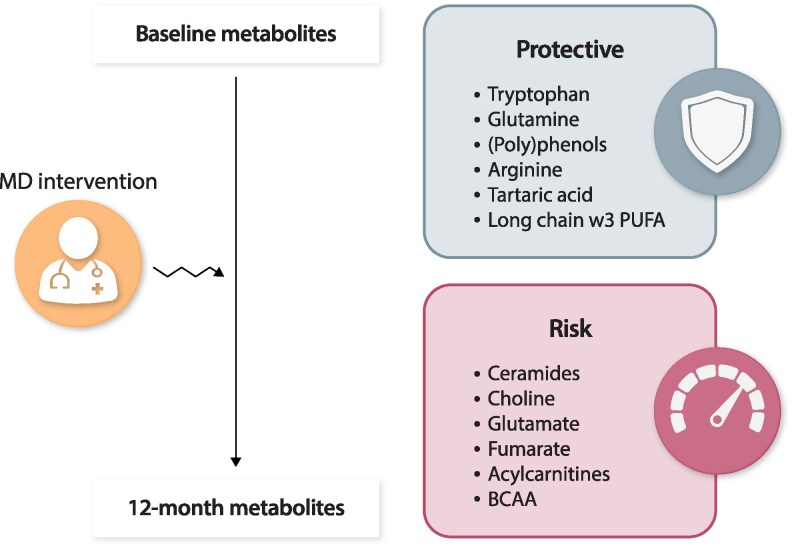
Metabolites related to the Mediterranean diet and CVD. MD: Mediterranean Diet. PUFA: polyunsaturated fatty acids. BCAA: branched-chain amino acids.

The application of metabolomics in nutrition allows the identification of multi-metabolomic signatures, which integrate the profiles of multiple metabolites and may allow the capture of overall dietary patterns. These signatures reflect both dietary intake and the homeostatic responses of the organism to the exposure to specific foods. Thus, metabolomic signatures of dietary patterns, such as the MedDiet, allow the measurement of adherence to dietary patterns. Multi-metabolite signatures can capture the entire dietary pattern and predict long-term major cardiovascular events. In fact, several metabolomics studies nested in PREDIMED have assessed metabolic pathways, and they contributed to a more complete understanding of the mechanisms involved in the long-term prevention of cardiometabolic events. Particularly, a multi-metabolite signature for the MedDiet, consisting of 67 plasma metabolites measured at baseline, predicted the long-term incidence of the primary cardiovascular endpoint (myocardial infarction, stroke or cardiovascular death). These findings were also replicated in three US cohorts. Several case-cohort and case-control studies nested in the PREDIMED study were included in a recent narrative review^72^, and they are briefly summarized in *Figure 5*. Some examples of potentially harmful effects related to metabolic pathways that were shown to be beneficially modified by MedDiet interventions and can account for the long-term reductions in cardiometabolic health events are changes in plasma glutamine concentrations, which were associated with a lower risk of CVD. In contrast, glutamate predicted an increased cardiovascular risk.^73^ Plasma tryptophan was associated with a lower cardiovascular risk, which may explain the protective effect of extra-virgin olive oil in the MedDiet.^74,75^ Acylcarnitines, leucine, isoleucine and plasma ceramides were associated with an increased risk of cardiovascular events.^76–78^ Increased availability of total arginine and cholesteryl esters were inversely associated with CVD risk,^79–83^ whereas plasma choline and some citric acid cycle metabolites were associated with an increased risk of CVD.^84–86^

The MedDiet interventions were able to modify the lipidome in the PREDIMED trial.^81^ Lipid metabolites with a longer acyl chain and a higher number of double bonds at baseline were significantly and inversely associated with the risk of CVD. Using factor analysis to identify lipid classes associated with type 2 diabetes (T2D) in patients at high cardiovascular risk, a profile including Lysophospholipids (LPs), Phosphatidylcholine-Plasmalogens (PC-PLs), Sphingomyelins (SMs), and Cholesteryl Esters (CEs) was associated with lower T2D risk. Another profile composed of Triacylglycerols (TAGs), Diacylglycerols (DAGs), and Phosphatidylethanolamines (Pes) was associated with higher T2D risk.^87,88^

## 11. Conclusion

In conclusion, the MedDiet has been established as the most intensely studied and the best scientifically proven dietary pattern for the prevention of cardiovascular disease. Major clinical trials such as the Lyon Diet Heart Study, PREDIMED, PREDIMED-Plus, and CORDIOPREV have provided compelling evidence that adherence to the MedDiet, characterized by a high consumption of fruits, vegetables, extra-virgin olive oil, nuts, legumes, and low-to-moderate amounts of red wine, is associated with a lower long-term risk of cardiovascular events.

Studies of metabolomics have improved our understanding of the mechanisms underlying the benefits of the MedDiet. They have also allowed the identification of biomarkers that could help to monitor adherence to this dietary model, and they have opened new knowledge with translations for precision nutrition and personalized disease prevention.

On the other hand, the moderate consumption of red wine with meals and its beneficial effects on cardiovascular health is a much-debated aspect of the MedDiet. A new large non-inferiority randomized trial, UNATI, will help to clarify the doubts about the potential beneficial effect of including or removing wine from the MedDiet.

In summary, the MedDiet remains an ideal dietary model with the strongest scientific evidence for its effects on the prevention of chronic diseases such as cardiovascular disease.^89^ Despite the limitations of some studies, their beneficial effects are consistent and relevant to global public health.
